# Unusual manifestations of primary Glioblastoma Multiforme: A report of three cases

**DOI:** 10.4103/2152-7806.74146

**Published:** 2010-12-22

**Authors:** Ahmet Metin Sanli, Erhan Turkoglu, Habibullah Dolgun, Zeki Sekerci

**Affiliations:** Ministry of Health Diskapi Yildirim Beyazit Education and Research Hospital, 1^st^ Neurosurgery Clinic, 06110 Ankara, Turkey

**Keywords:** Astrocytoma, brain tumor, glioblastoma multiforme, presentation, symptom

## Abstract

**Background:**

Brain tumors, especially high-grade gliomas, can present with focal or generalized signs due to mass effect, parenchymal infiltration and destruction. In general, at the time of diagnosis, tumors could cause common neurological symptoms and major clinical signs depending on their localization. In rare instances, brain tumors colud be manifested with unusual symptoms.

**Case Description:**

We describe three cases presenting with unusual clinical symptoms: ulnar neuropathy, vertigo and syncope attacks. Microscopic total tumor excision was done and histopathological analysis revealed that these tumors were glioblastoma multiforme. Both external beam radiotherapy and chemotherapy were given as adjuvant treatments.

**Conclusions:**

Physicians should keep brain tumors in mind in the case of patients who present with atypical symptoms such as those reported here. Brain imaging should be performed over a prolonged period following presentation if the patient’s symptoms remain unresolved after adequate treatment.

## INTRODUCTION

Astrocytomas, including glioblastoma multiforme (GBM), are the most common malignant central nervous system (CNS) tumors in neurosurgical practice.[5] GBM is the most common and aggressive type of primary brain tumor in humans, accounting for 52% of all primary brain tumor cases and 20% of all intracranial tumors.[12] GBM can present with focal or generalized signs due to mass effect, parenchymal infiltration and destruction.[1,3] Depending on their localization, GBMs often cause common neurological symptoms and major clinical signs at the time of diagnosis. The former include headache (56%), vomiting and nausea (13%), seizures (32%), and cognitive dysfunction (34%); the latter include focal neurological deficits (in approximately 23% of cases) such as gait disturbance, disorientation, and cranial nerve dysfunction.[8,9,13] On the other hand, GBMs not associated with common neurological symptoms and signs are unusual. In rare instances, patients with brain tumors can manifest predominantly with vertigo, unusual focal neurological signs such as dermatomal hypoesthesia or psychiatric symptoms.[13] In the light of these unusual symptoms, physicians could err in their diagnosis and formulate treatment plans, including surgical and medical interventions, without considering the possibility of CNS tumors. Accurate diagnosis in these cases can be extremely difficult. In this report, we describe three patients who presented with unusual clinical symptoms: ulnar neuropathy, vertigo and syncope attacks, respectively.

## CASE REPORTS

### Case 1

**Figure 1:**
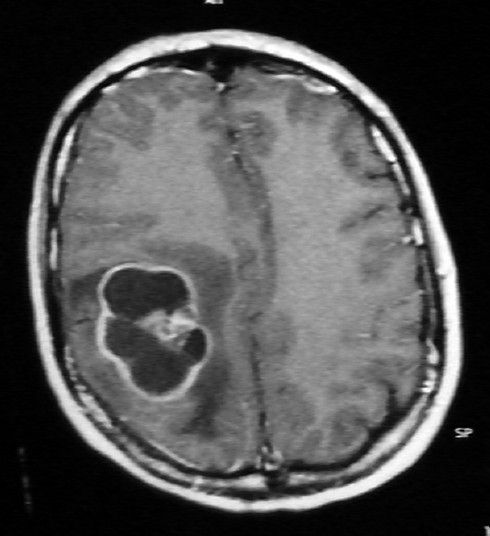
Cranial axial MRI with gadolinium revealing a ring-enhancing 4 × 4.5 cm mass lesion in the right parietal lobe with significant surrounding peripheral edema.

A 42-year-old man was admitted with progressive tingling, paresis and hypoesthesia of his left hand through an orthopedic clinic. Electromyography and nerve conduction studies revealed that the velocity of the ulnar nerve was slowed between the elbow and 2 cm site above of elbow [Table 1], even though his neurological findings were normal. He was diagnosed with left ulnar nerve entrapment neuropathy at the elbow; accordingly, left ulnar nerve decompression and anterior transposition surgery was performed. However, the orthopedic neurosurgeon had noticed during the surgery that entrapment was not manifested according to EMG and his symptoms did not improve after this surgical treatment. Seven days after discharge, he applied to our clinic with sensory seizures on his left arm and left hemifacial region with the thought that the symptoms were not truly consistent with ulnar nerve entrapment. Neurological examination also revealed that left upper extremity was plegic, and his left lower extremity exhibited +4/5 motor strength. Immediate cranial computed tomography (CT) without contrast revealed a large heterogeneous hypodense area involving the right basal ganglia, frontoparietal cortex and white matter causing a minimal midline shift. He was hospitalized and started on phenytoin (300 mg/day, IV), dexamethasone (16 mg/day, IV), and mannitol (1 mg/kg, IV). Cranial magnetic resonance imaging (MRI) with gadolinium demonstrated a ring-enhancing 4 × 4.5 cm mass lesion in the right parietal region with significant surrounding peripheral edema [Figure 1]. Microscopic total tumor excision was done and histopathologic analysis revealed that the tumor was GBM. Both external beam radiotherapy (RT) and chemotherapy were given as adjuvant treatments, but the patient died 12 months following the initial diagnosis.

**Table 1:** Motor nerve conduction study of left ulnar nerve

	Sites	Latency (ms)	Amp.2-4 mV	Distance (cm)	Velocity (m/s)
L. Ulnar	Wrist	2.15	9.6		
	B. Elbow	5.70	8.9	20	56.3
	A. Elbow	7.85	8.2	10	46.5
R. Ulnar	Wrist	2.25	14.0		
	B. Elbow	5.80	13.3	22	62.0
	A. Elbow	7.65	12.4	10	54.1
Inching	Site 1	7.95	7.7		
	Site 2	7.70	7.8		
	Site 3	7.35	8.1		
	Site 4	6.15	8.0		
	Site 5	5.85	8.0		
	Site 6	5.70	8.0		

The ulnar nerve is divided into six regions each with 2 cm with reference to olecranon. Nerve conduction velocity and latency of left ulnar nerve decelareted are compared with right ulnar nerve. These findings indicated left ulnar entrapment neuropathy.

A; above, Amp; amplitude, B; below, cm; centimetre, L; left, ms; millisecond, mV; milivolt, R; right

### Case 2

**Figure 2:**
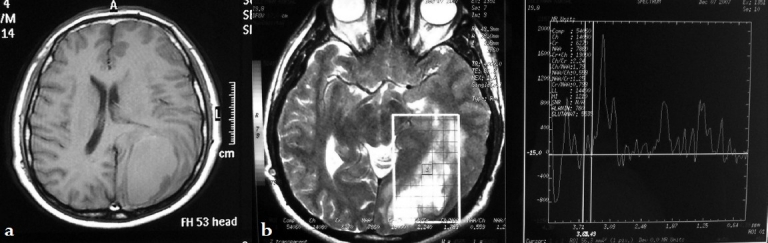
(a) T1-weighted axial cranial MRI without contrast revealing a hypointense lesion in the left parieto-occipital region causing a minimal midline shift (b) MR spectroscopy demonstrated an increased lactate peak, decreased N- acetylaspartate (NAA) peak and creatinin (Cr). These findings suggest that the appearance could be geared to acute intracerebral hematoma

**Figure 3:**
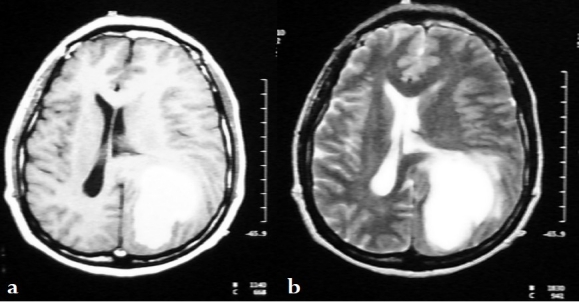
(a, b) Cranial MRI with gadolinium revealing a 6 × 5 × 4 cm left-sided parieto-occipital lesion with brain edema, associated mass effect and uncal herniation. The lesion was composed of cystic tissue separated by various fibrous septae. The tumor was hyperintense on T1-(a) and T2-(b) weighted images and showed heterogeneous enhancement with contrast peripherally.

A 46-year-old man presented with multiple syncope attacks. The patient felt faint and then passed out. The physician did not think that these drop attacks were secondary to seizure activity. Six months earlier, he had begun to notice intermittent headaches. He applied to our emergency department with confusion, a severe headache and left side hemiparesis and hemihypoesthesia. He was referred to a neurology clinic, and a right parieto-occipital intracerebral hematoma was detected on cranial MRI. Our differential diagnosis included intracranial malignancies,[4] so cranial MRI, diffusion MRI, and magnetic resonance spectroscopy (MRS) were performed. T1- weighted non-contrast axial cranial MRI scan demonstrated a lesion in the left parieto-occipital region causing a minimal midline shift. MRS demonstrated an increased lactate peak, a decreased *N*- acetylaspartate peak and creatinine [Figures 2a and b]. The diffusion-weighted MRI showed no diffusion restriction in B1000 images. These findings indicated that the lesion was a hematoma. The patient was hospitalized and started on medical therapy for an intracerebral hematoma even if this was not a common place for hypertensive intracerebral hematoma. Eventually, he was discharged without any sequelae. After two weeks, the patient was again admitted to the emergency department with another attack of syncope. Immediate cranial CT revealed a 6 × 5 × 4 cm hypodense lesion causing a midline shift with significant peripheral edema in the left parieto-occipital area. The patient was again hospitalized and started on dexamethasone (16 mg/day, p.o) and mannitol (1gr/kg, IV). Cranial axial MRI with gadolinium showed a 6 × 5 × 4 cm left-sided parieto-occipital lesion with brain edema, associated mass effect and uncal herniation. The tumor was hyperintense on T1- and T2- weighted images and showed heterogeneous enhancement with contrast peripherally [Figures 3a and b]. The tumor was removed with the use of an operative microscope. Histopathological examination revealed that the lesion was a GBM. Both external beam RT and chemotherapy were given as adjuvant treatments. The patient is still alive and is currently without any neurological sequelae.

### Case 3

**Figure 4:**
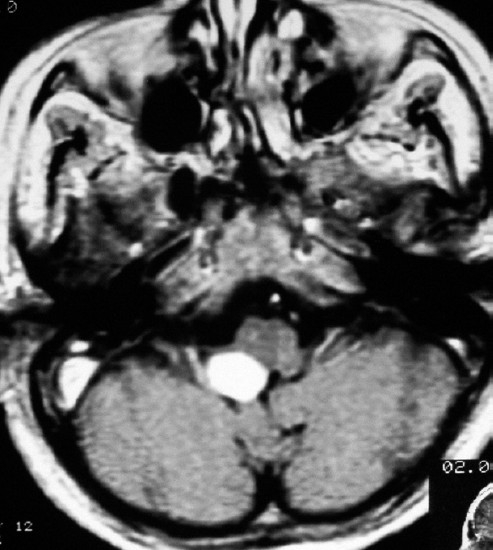
Cranial axial MRI with contrast showing a 1.5 × 2 cm nodular tumor, localized in the right lateral recess of the forth ventricle and showing homogenous enhancement.

A 70-year-old woman presented with progressive and continuous vertigo for six months. She had complained about right-sided otalgia and bilateral hearing loss without tinnitus and applied to the ear, nose, and throat (ENT) clinic. The patient had a normal ear examination but audiometry demonstrated 60-70 dB moderate sensorineuronal hearing loss bilaterally. Neurological examination was normal except left-sided nystagmus. Cranial axial MRI with contrast revealed a 1.5 × 2 cm nodular tumor in the right lateral recess of forth ventricle [Figure 4]. The tumor was hypointense on T1-weighted and hyperintense on T2-weighted images and showed strong homogenous enhancement. Surgery was performed via a suboccipital approach. The tumor originated from the right dorsal side of the brainstem and was completely removed. Histopathological examination revealed that the lesion was GBM. Both external beam RT and chemotherapy were given as adjuvant treatments. The patient died four months later following the diagnosis.

## DISCUSSION

Pre-operative evaluation of patients with possible brain lesions requires a careful history and physical examination followed by radiological examination. Patients with high-grade gliomas (HGG) often present with headache, new-onset seizures, altered mental functions[16] (e.g. personality changes), nausea, vomiting or focal neurological signs and symptoms [Table 2]. Neurological signs and symptoms vary depending on tumor location and size. While small tumors in eloquent cortical regions or the brainstem may cause significant focal deficits, large tumors in the frontal lobe may present only minor personality alterations.[1] Headache is the most common initial presenting symptom and is usually associated with elevated intracranial pressure.[12] One-third of patients with HGG present with nausea, vomiting and visual disturbances, mostly in association with headaches.[8] Seizures are most common in patients with low-grade gliomas. Patients with GBM can also present with seizures as an initial symptom.[17] The type of seizure may vary with tumor location, and the seizures can be focal or generalized. The other most common symptom is cognitive dysfunction. Changes in memory, attention, orientation, personality, language ability, executive functions and daily activities are the commonly seen dysfunctions mainly associated with tumors that originate from the dominant hemisphere.[3]

**Table 2:** Common presenting signs and symptoms in patients with high-grade brain tumors

Signs and symptoms	Percentage
Headache	56
Memory loss	35
Cognitive changes	34
Motor deficit	33
Language disorder	32
Seizures	32
Personality changes	23
Visual problems	22
Changes in consciousness	16
Nausea or vomiting	13
Sensory deficit	13
Papilledema	5

Patients with primary GBM characteristically present with focal or generalized symptoms as mentioned above. Because GBM is the most common primary brain tumor, there are many instances of unusual presentations. These including, but are not limited to unusual seizures patterns (e.g. musicogenic epilepsy, reflex epilepsy),[6,10,14] unusual language problems (e.g. pseudo-foreign language syndrome),[7,15] unusual pain syndrome (e.g. various type of allodynia secondary to thalamic or primary sensory cortex invasion),[15] and unusual personality changes (e.g. hypersexuality), and anorexia nervosa.[2,7,11,13] In rare instance, the patients can manifest syncope attacks, vertigo, vestibular neuritis and local signs such as sensory seizures and/or entrapment neuropathy similar to our cases. Accurate diagnosis in these cases can be extremely difficult. In light of these unusual symptoms, physicians could err in their diagnoses and treat patients with surgical and medical interventions that do not account for the possibility of CNS tumors.

Despite the attempts to classify symptoms as focal or generalized and to determine GBM locations, clinical situations are nonspecific and unreliable. Therefore, physicians should keep brain tumors in mind in the case of patients who present with atypical symptoms such as those reported here. Consequently, brain imaging should be performed over a prolonged period following presentation if the patient’s symptoms remain unresolved after adequate treatment. High suspicion of GBM is mandatory in patients over 40 years of age who present with changes in neurological status.

## References

[1] Bucker JC, Brown PD, O’Neill BP, Meyer FB, Wetmore CJ, Uhm JH (2007). Central nervous system tumors. Mayo Clin Proc.

[2] Burns JM, Swerdlow RH (2003). Right orbitofrontal tumor with pedophilia symptom and constructional apraxia sign. Arch Neurol.

[3] Chandana SR, Movva S, Arora M, Singh T (2008). Primary brain tumors in adults. Am Fam Physician.

[4] Chang SM, Parney IF, Huang W, Anderson FA, Asher AL, Bernstein M (2005). Patterns of care for adults with newly diagnosed malignant glioma. JAMA.

[5] Department of Health and Human Services, Centers for Disease Control and Prevention (CDC), National Program of Cancer Registries (NPCR). Central brain Tumor Registry of the United States.

[6] El Bouzidi K, Duncan S, Whittle IR, Butler CR (2010). Lesional reflex epilepsy associated with the thought of food. Neurology.

[7] Filley CM, Kleinschmidt-DeMasters BK (1995). Neurobehavioral presentations of brain neoplasms. West J Med.

[8] Forsthy PA, Posner JB (1993). Headache in patients with brain tumors: A study of 111 patients. Neurology.

[9] Frankel SA, German WJ (1958). Glioblastome multiforme: A review of 219 cases with regard to natural history, pathology, diagnostic methods, and treatment. J Neurosurg.

[10] Green JB (1984). Pilomotor seizures. Neurology.

[11] Lin L, Liao SC, Lee YJ, Tseng MC, Lee MB (2003). Brain tumor presenting as anorexia nervosa in a 19-year-old man. J Formos Med Assoc.

[12] Louis DN, Ohgaki H, Wiestler OD, Cavenee WK, Burger PC, Jouvet A (2007). The 2007 WHO Classification of Tumors of the central nervous system. Acta Neuropathol.

[13] Madhusoodanan S, Danan D, Moise D (2007). Psychiatric manifestations of brain tumors: Diagnostic implications. Expert Rev Neurother.

[14] Marchini C, Romito D, Lucci B, Del Zotto E (1994). Fits of weeping as an unusual manifestation of reflex epilepsy induced by speaking: Case report. Acta Neurol Scand.

[15] Ojemann JG, Miller JW, Silbergeld DL (1996). Preserved function in brain invaded by tumor. Neurosurgery.

[16] Rostomily RC, Spence AM, Silbergeld DL, Moore AJ, Newell DW (2004). Neurosurgical Management of high grade gliomas. Chap 10. International Practices in Neurosurgery.

[17] Roth JG, Elvide AR (1960). Glioblastome multiforme: A clinical survey. J Neurosurg.

